# *Let*-*7* as biomarker, prognostic indicator, and therapy for precision medicine in cancer

**DOI:** 10.1186/s40169-019-0240-y

**Published:** 2019-08-28

**Authors:** Evgeny Chirshev, Kerby C. Oberg, Yevgeniya J. Ioffe, Juli J. Unternaehrer

**Affiliations:** 1 Division of Anatomy, Department of Basic Sciences, Loma Linda University, Loma Linda, CA USA; 2 Division of Anatomy and Pediatric Pathology, Loma Linda University, Loma Linda, CA USA; 3 Gynecology and Obstetrics, School of Medicine, Loma Linda University, Loma Linda, CA USA; 4 Division of Biochemistry, Department of Basic Sciences, Loma Linda University, 11085 Campus Street, Mortensen Hall 219, Loma Linda, CA 92354 USA

**Keywords:** microRNA, Cancer, Gene regulation, Biomarker, Therapeutics, Tumor suppressor

## Abstract

Abnormal regulation and expression of microRNAs (miRNAs) has been documented in various diseases including cancer. The miRNA *let*-*7* (MIRLET7) family controls developmental timing and differentiation. *Let*-*7* loss contributes to carcinogenesis via an increase in its target oncogenes and stemness factors. *Let*-*7* targets include genes regulating the cell cycle, cell signaling, and maintenance of differentiation. It is categorized as a tumor suppressor because it reduces cancer aggressiveness, chemoresistance, and radioresistance. However, in rare situations *let*-*7* acts as an oncogene, increasing cancer migration, invasion, chemoresistance, and expression of genes associated with progression and metastasis. Here, we review *let*-*7* function as tumor suppressor and oncogene, considering *let*-*7* as a potential diagnostic and prognostic marker, and a therapeutic target for cancer treatment. We explain the complex regulation and function of different *let*-*7* family members, pointing to abnormal processes involved in carcinogenesis. *Let*-*7* is a promising option to complement conventional cancer therapy, but requires a tumor specific delivery method to avoid toxicity. While *let*-*7* therapy is not yet established, we make the case that assessing its tumor presence is crucial when choosing therapy. Clinical data demonstrate that *let*-*7* can be used as a biomarker for rational precision medicine decisions, resulting in improved patient survival.

## Introduction

**Fig. 1:**
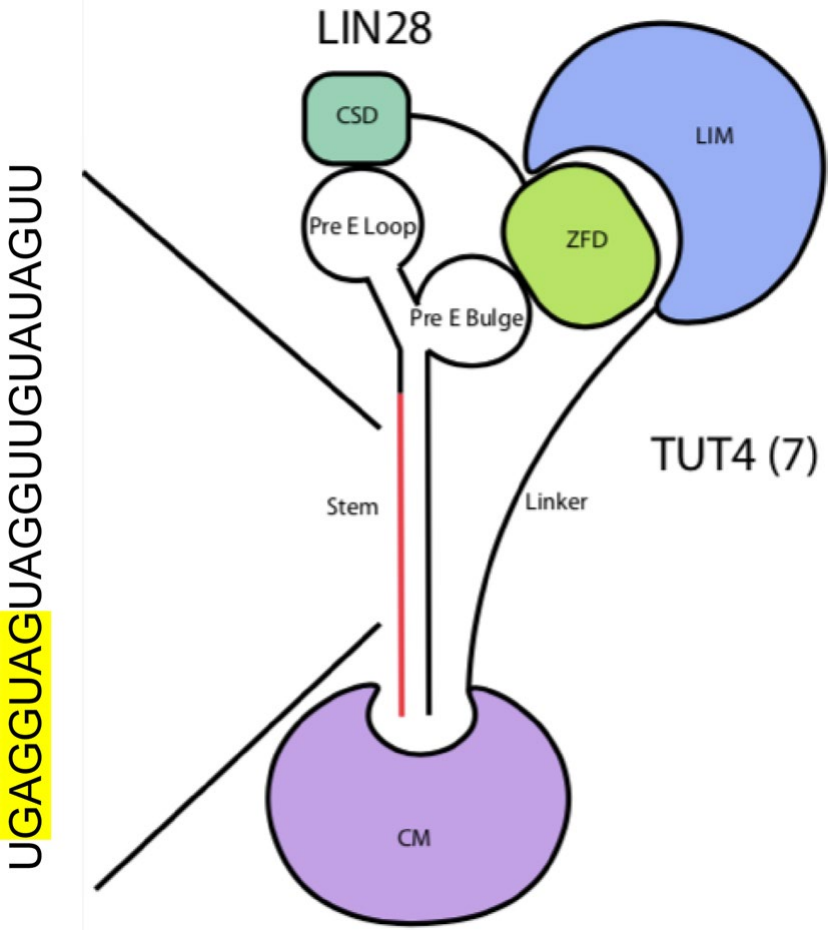
LIN28/TUT4(7) *let*-*7* binding during post-transcriptional processing. Modified from Nam et al. and Faehnle et al. [111, 112]. CSD and ZFD are abbreviations for cold shock domain and zinc finger domain of LIN28 respectively. LIM and CM are abbreviations of LIN28 interactive module and catalytic module of TUT4(7) respectively. Sequence of mature *let*-*7a* demonstrates position of let-7 family members in the stem along with highlighted seed region

During carcinogenesis, cells acquire capabilities termed the hallmarks of cancer [1]. Abnormal microRNA (miRNA) regulation has been attributed to all phases of cancer and affects several of the cancer hallmarks [2, 3]. Discovered in *C*. *elegans*, *let*-*7* (lethal-7) miRNA family functions as an important regulator of differentiation [4, 5]. In mammals, *let*-*7* is known as the keeper of differentiation, and its abnormal regulation and expression has been associated with cancer initiation and progression [6]. The functions of all members are generally thought to be overlapping because of sequence similarity [7]. Figure 1 includes a diagram of *let*-*7* structure with seed sequence highlighted. Because *let*-*7* targets several oncogenes, its repression in cancer is most often associated with poor patient prognosis [8]. The human genome contains 13 *let*-*7* family members encoding 9 mature miRNAs. *Let*-*7a1*, *a2*, *a3* are encoded from different transcripts, producing identical mature sequence; the same is true for *let*-*7f1*, *f2*. With the exception of *let*-*7i* and *let*-*7g*, which are encoded individually, transcripts of different *let*-*7* members are located in clusters along with other miRNAs [9–12]. Due to different genomic loci, transcriptional regulation varies between individual *let*-*7* family members. In this review, we discuss *let*-*7*′s involvement in patient survival, focusing on its function as diagnostic, prognostic and therapeutic, in isolation as well as in combination with current therapy regimens. We review *let*-*7* effects on cellular phenotype, and explain it by molecular mechanisms. We also discuss instances of *let*-*7* oncogenic functions, differences between regulation and function of different *let*-*7* family members, and its importance for understanding its effect in cancer biology and its therapeutic potential.

## *Let*-*7* as tumor suppressor

### Use as a screening tool

Levels of *let*-*7* family members can serve as biomarkers to assist with cancer diagnosis, and monitoring. Detecting differential *let*-*7* levels in bodily fluids has the potential to allow early detection of cancer using minimally invasive procedures, minimizing risks associated with biopsy. Increased plasma *let*-*7* levels are seen in patients with breast, prostate, colon, renal, liver, gastric, thyroid, and ovarian cancer [13–19]. Elevated urine *let*-*7* levels can also be detected in renal cancer [20]. Some studies have also reported decreased serum *let*-*7* levels in colon, lung, prostate, gastric, ovarian, and breast cancers [21–29]. Patients with colorectal carcinoma have decreased levels of *let*-*7* in stool samples compared to healthy controls, providing a less invasive tool to aid with diagnosis [22]. These studies, with some apparently contradictory results, point out the need for further study, but the use of serum *let*-*7* appears to be a promising biomarker. For each cancer type, results are consistent. Table 1 provides a summary of abnormal *let*-*7* levels in plasma (liquid biopsy) based on the type of cancer. Plasma *let*-*7* levels have the potential to serve as a monitoring system for therapy, and may predict accelerated reproliferation of lung cancer, which would assist in providing personalized treatment options for patients [30]. *Let*-*7* levels are directly influenced by therapy, illustrated by *let*-*7c* in acute promyelocytic leukemia: its levels increase in blasts after chemotherapy, then decrease again upon relapse [31]. Chemoresistant epithelial ovarian cancers, lung cancers, and acute myeloid leukemia have reduced *let*-*7* levels relative to chemo-sensitive cells, resulting in non-response to chemotherapy [32–37]. These are examples of ways that monitoring *let*-*7* levels could be used to predict drug response or recurrence. Thus, much more work remains in order to understand the diagnostic value of *let*-*7* levels in blood and urine.

**Table 1:** Levels of serum *let*-*7* relative to normal control in patients with different types of cancer

*Let*-*7* expression in liquid biopsy		
Cancer	Increased	Decreased
Breast	*let*-*7a* [13] *let*-*7b* [16, 17] *let*-*7c* [17] *let*-*7i* [17]	*let*-*7c* [28]
Prostate	*let*-*7a* [13]	*let*-*7a* [24]
Colon	*let*-*7a* [13, 14]	*let*-*7a* [21, 22] *let*-*7b* [21] *let*-*7c* [21] *let*-*7f* [22] *let*-*7i* [21]
Renal	*let*-*7a* [13]	
Lung		*let*-*7a* [23] *let*-*7b* [23] *let*-*7c* [29] *let*-*7f* [26] *let*-*7i*^a^ [15]
Gastric	*let*-*7f* [19] *let*-*7i* [19]	*let*-*7a* [25]
Liver	*let*-*7b* [15] *let*-*7f* [18]	
Ovarian	*let*-*7b* [134]	*let*-*7f* [27] *let*-*7i* [135]
Thyroid	*let*-*7e* [136]	

In these experiments, liquid biopsies (from blood) were sampled

^a^Associated with smoking

### Use as a diagnostic tool for therapy selection

*Let*-*7* is repressed in many different types of human cancer. Table 2 summarizes abnormal *let*-*7* expression obtained from patient tumors, obtained by solid biopsy, and cultured cancer cell lines (including cases where *let*-*7* is up-regulated). Mechanisms for loss of *let*-*7* are incompletely understood, however studies in ovarian cancer suggest that *let*-*7* repression is due to genomic deletions and abnormal transcription, rather than loss of processing mechanisms involving Dicer and Drosha [38]. While 31.2% of epithelial ovarian cancers (EOC) demonstrate *let*-*7a3* deletions, only 3.1% had alterations in copy number of *let*-*7i* [36, 38]. Abnormal expression of *let*-*7* family members correlates with patient prognosis. Decreased expression of *let*-*7* correlates with aggressive, high-grade tumors, and poor prognosis; accordingly, high *let*-*7* levels are associated with better prognosis and prolonged patient survival [36, 39–46]. Low post-surgical tumor *let*-*7* levels indicate poor prognosis for lung cancer patients, with reduced overall survival [44, 47]. The picture is similar for breast, pancreatic, colorectal, liver, and ovarian cancer (the only exception is *let*-*7b* and *c* in ovarian cancer) [17, 36, 39–43, 45, 46]. Of note, high *let*-*7b* levels in high grade serous EOC positively correlate with markers of invasiveness and worse prognosis. Let-7 family members are expressed at lower levels in metastatic sites compared to primary tumor in gastric, breast, liver, and lung cancers [48–51]. In vivo, let-7 over-expression in breast cancer resulted in reduced lung and liver metastasis, while let-7 repression resulted in increased in metastasis [51, 52]. Therefore, tumor let-7 levels correlate with and can be used as a prediction for distal metastasis. Thus, while important exceptions must be noted, loss of *let*-*7* in most cancers closely correlates with poor prognosis.

**Table 2:** Levels of *let*-*7* family members relative to normal tissue in different types of human cancer

*Let*-*7* expression in tumors		
*Let*-*7*	Decreased	Increased
*Let*-*7a*	Hepatoblastoma [70] Glioma [67] Ewing sarcoma [85] Gastric [48] Nasopharyngeal [70] Lung [47] Liver [42, 96] Melanoma [75] Endometrial [137] Cervical [107, 128] Prostate [138, 139] Ovarian [43]	
*Let*-*7b*	Hepatoblastoma [66] Liver [42, 96] Melanoma [75] Prostate [138, 139]	Ovarian [43]
*Let*-*7c*	Prostate [58, 139] Acute promyelocitic leukemia [31] Liver [42, 96] Lung [44] Endometrial [137] Prostate [138]	Ovarian [43]
*Let*-*7d*	Oral [62] Liver [42] Melanoma [75] Prostate [138] Ovarian [140]	Acute promyelocytic leukemia [31]
*Let*-*7e*	Melanoma [75] Endometrial [137] Prostate [138] Ovarian [140]	Tongue [99] Esophageal [97]
*Let*-*7f*	Liver [42] Endometrial [137] Prostate [138] Ovarian [43, 140]	Tongue [99]
*Let*-*7* *g*	HCC [42] Prostate [138] Ovarian [43]	
*Let*-*7i*	Tongue [99] Ovarian [141] Melanoma [75] Cervical [107]	
*Mir*-*98*	Glioma [71] Salivary adenoid cystic carcinoma [72] Prostate [138]	Ovarian [100]

In these experiments, tumors were sampled

While *let*-*7* levels in body fluids can possibly assist in diagnosis, *let*-*7* levels in tumors can be used to create a personalized optimal treatment plan including both chemotherapy and radiation. Determining levels of tumor *let*-*7* as well as its targets is expected to be useful to deliver personalized treatment when considering therapy options. Colorectal carcinoma patients with KRAS mutation and with high levels of *let*-*7* can benefit from anti-EGFR therapy, while patients with low levels of *let*-*7* have impaired responses [45]. A study by Lu et al. examined *let*-*7a* expression levels and response to chemotherapy in patients treated with platinum-based chemotherapy with or without paclitaxel. The patients in this study were treated between 1991 and 2000 [53]. The platinum/paclitaxel doublet became the first line standard of care in advanced EOC after the publication of results of GOG 111 in 1996 [54]. In the study by Lu and colleagues, patients with ovarian cancer and high *let*-*7a* levels have better prognosis than those with low *let*-*7a* levels when treated with platinum-based therapy alone, but counterintuitively, high *let*-*7a* levels correlate with poor response when platinum is combined with paclitaxel. The reverse was true for patients with low *let*-*7a* levels in tumors [53]. These observations lead to the hypothesis that, for patients with high tumor *let*-*7a* levels, forgoing paclitaxel results in improved outcomes. Paclitaxel inhibits microtubule polymerization, thus affecting rapidly dividing cells. *Let*-*7* has anti-proliferative functions (described below), providing a possible explanation why patients with high tumor *let*-*7* levels did not respond well to paclitaxel [55]. Therefore, knowledge of tumor *let*-*7a* levels is expected to be an important contributor to decisions about chemotherapy. However, this will require careful consideration and further retrospective trials followed by robust clinical trials, as currently doublet platinum based or combination intravenous/intraperitoneal chemotherapy are recommended for advanced ovarian cancer in the front line setting (NCCN Guidelines Ovarian Cancer Version 2.2018 3/14/2018, http://www.nccn.org, accessed 1/6/2019).

However, what is true for ovarian cancer may not apply to other malignancies: breast cancer cell lines that have low *let*-*7* levels respond better to taxol treatment [56]. The discrepancy between results obtained by the studies in ovarian and breast cancer can be attributed to differences in biology of breast and ovarian cancer as well as the study design. While Lu et al. [53] compared clinical data and tumor *let*-*7a* levels from ovarian cancer patients that had undergone different courses of treatment, Sun et al. [56] used breast cancer cell lines for in vitro studies. Tumor *let*-*7* levels can also predict response to other therapies. Breast cancer patients with low tumor *let*-*7* levels do not respond to epirubicin; therefore, choosing an alternative therapy may prolong survival [57]. In prostate cancer patients, tumors with decreased *let*-*7c* levels are resistant to androgen therapy, and *let*-*7* delivery to tumors provides promising therapy [58]. Table 3 summarizes best therapy options based on *let*-*7* levels in several types of cancer.

**Table 3:** Therapy of choice based on tumor *let*-*7* levels in different types of cancer

Rational therapy choice based on *let*-*7* expression			
Cancer	*Let*-*7* levels	Additional	Therapy of choice
Colorectal	High	KRAS mutation	Anti-EGFR therapy [45]
Ovarian	High		Platinum [53]
	Low		Platinum with Paclitaxel [53]
Breast	Low		Taxol [56] No response to Epirubicin [57]
Prostate	Low		Resistant to androgen therapy [58]

### *Let*-*7* replacement as a therapeutic

Tumor delivery of *let*-*7* is a potential therapy, as a strategy for reversing stemness and chemoresistance, in combination with chemotherapy [59]. *Let*-*7* over-expression results in increased sensitivity to chemotherapy and radiation therapy in ovarian cancer, hepatocellular carcinoma, oral squamous carcinoma, breast cancer, lung cancer, and myeloid leukemia, while inhibiting *let*-*7* results in acquisition of resistance [35–37, 57, 59–65].

Higher tumor *let*-*7* levels contribute to an increase in sensitivity to therapy [36, 57, 58]. This decrease in resistance can allow treatment with lower dose of chemotherapy to obtain the same therapeutic benefit. This represents an opportunity to avoid severe side effects of cancer treatments by using lower chemotherapy dosages. The ability to use lower drug dosages to obtain equivalent therapeutic benefit may lead to lower levels of toxicities and chemotherapy related adverse events, allowing for better quality of life for the patients undergoing treatment. Also, there would likely be fewer instances of chemotherapy discontinuation due to lower instances of dose-limiting toxicities.

The feasibility of using *let*-*7* as therapy has been demonstrated by successful in vivo studies. *Let*-*7* overexpression in animal model studies results in reduction of tumor size, metastasis, and prolonged survival [35, 42, 52, 58, 59, 62, 66–68]. These results are explained via functional assays in vitro, where *let*-*7* decreases cellular proliferation, migration, and invasion [37, 42, 58, 59, 67–73]. *Let*-*7* overexpression has been accomplished in pre-clinical murine models via *let*-*7* mimics, demonstrating its efficacy. As miRNA will rapidly degrade in plasma, advanced *let*-*7* delivery methods are required. In order to avoid tissue toxicity and delivery to other cells within tumor stroma, strategies for delivery of mimics specifically to cancer cells must be developed (see below). Dai et al. utilized polyethyleneglycol (PEG) nanoparticles to deliver *let*-*7* together with paclitaxel in vivo, and they observed successful repression of tumor burden without animal toxicity [59].

## Molecular aspects governing functional phenotype

The pleiotropic effects of *let*-*7* include repression of oncogenes, suppression of epithelial-to-mesenchymal transition, induction of chemosensitivity, controlling cell signaling pathways, and decreasing cellular proliferation.

*Let*-*7* effect on cancer observed in clinical, in vivo, and in vitro studies can be explained by several functional aspects. One way *let*-*7* acts as a tumor suppressor is via repression of oncogenes resulting in a decrease in stemness [60, 74]. *Let*-*7* levels inversely correlate with percentage of cancer stem cells (CSC), and its overexpression reduces CSC markers nestin and CD133 in glioblastoma and ALDH1 in breast cancer [40, 73]. To determine the presence of cancer stem cells functionally, spheroid (mammosphere in breast cancer) formation and colony formation assays are used. Spheroids are enriched for tumor initiating cells and have lower *let*-*7* levels, and in mammospheres that are allowed to differentiate, *let*-*7* levels increase [52]. *Let*-*7* over-expression inhibits stemness, resulting in reduced sphere formation [40, 52, 60, 64, 73]. Cancer cells with a stem-like phenotype are also able to form colonies, measured as clonogenicity. Up-regulation of *let*-*7* results in decreased clonogenicity [47, 58, 59, 75].

*Let*-*7* targets oncogenes and genes important for tumor initiation and progression including Myc, RAS, E2F1, E2F5, LIN28, ARID3B, PBX3, HMGA2 and long non-coding RNA *H*19 [42, 59, 70, 76]. Silencing these genes causes the functional tumor suppressive effects mediated by *let*-*7*. LIN28A is a well-known pluripotency marker that is present in embryonic stem cells (ESCs) and decreases upon differentiation [77]. In prostate cancer, LIN28 increases aggressiveness and results in increased tumor burden in vivo [78]. ARID3B and HMGA2 transcriptionally activate OCT-4 and SOX2, respectively, both of which are pluripotency factors highly expressed in ESCs [39, 67, 69, 70, 79–81]. Repression of H19 results in methylation of promoters of several other genes due to up-regulation of DNMT3b [37, 42, 48, 72, 73, 81, 82]. PBX3 is an oncogene that induces epithelial-to-mesenchymal transition and promotes invasiveness and metastasis of gastric cancer [42, 60, 70, 71, 83]. Thus, *let*-*7* can repress the function of a number of factors that can be recruited in oncogenesis. These examples illustrate the specific effects of *let*-*7* demonstrated to result in functional changes relevant to cancer.

Besides repression of oncogenes, *let*-*7* also plays a role in controlling cell signaling pathways. Overexpression of the *let*-*7* family member miR-98 results in reduced phosphorylation and down-regulation of Akt and Erk, which have been implicated in carcinogenesis [42, 59, 64, 72, 84]. In Ewing’s sarcoma, *let*-*7* directly represses signal transducer and activator of transcription 3 (STAT3) and results in a less aggressive cancer phenotype [85]. The STAT3 pathway regulates genes related to cell cycle and cell survival and is often linked to cancer progression. STAT3 activity correlates with chemo- and radioresistance and poor survival [86]. In breast cancer, *let*-*7* targets estrogen receptors, which activate WNT signaling and promote stemness and cancer aggressiveness [40]. *Let*-*7* down-regulates WNT signalling activity by targeting estrogen receptors in breast cancer and TCF-4 (a transcription factor downstream of WNT) in hepatocellular carcinoma. WNT pathway is a major regulator of cell proliferation, differentiation, and migration, and has been shown to promote tumor growth and contribute to cancer stem cell phenotype [60, 64, 87]. Cumulatively, *let*-*7′s* effects on cell signaling pathways impede the aggressive phenotype.

Another way that *let*-*7* exerts tumor suppressive effects is via inhibition of epithelial-to-mesenchymal transition (EMT). EMT is a normal process during embryonic development as well as wound healing. Cancer development and metastasis are associated with abnormal occurrence of EMT in somatic cells. During EMT, epithelial cells gain the ability to invade and metastasize [88]. Reduced *let*-*7* levels correlate with an increase in EMT markers Twist, Snail, vimentin, and N-cadherin, resulting in increased cancer aggressiveness, as assessed by spheroid formation, migration, invasion, mesenchymal appearance, and resistance to chemotherapy [44, 62, 72]. Over-expression of *let*-*7* reduces expression of Snail and N-cadherin, while increasing E-cadherin; these effects are proposed to be via HMGA2 [42, 59].

**Fig. 2:**
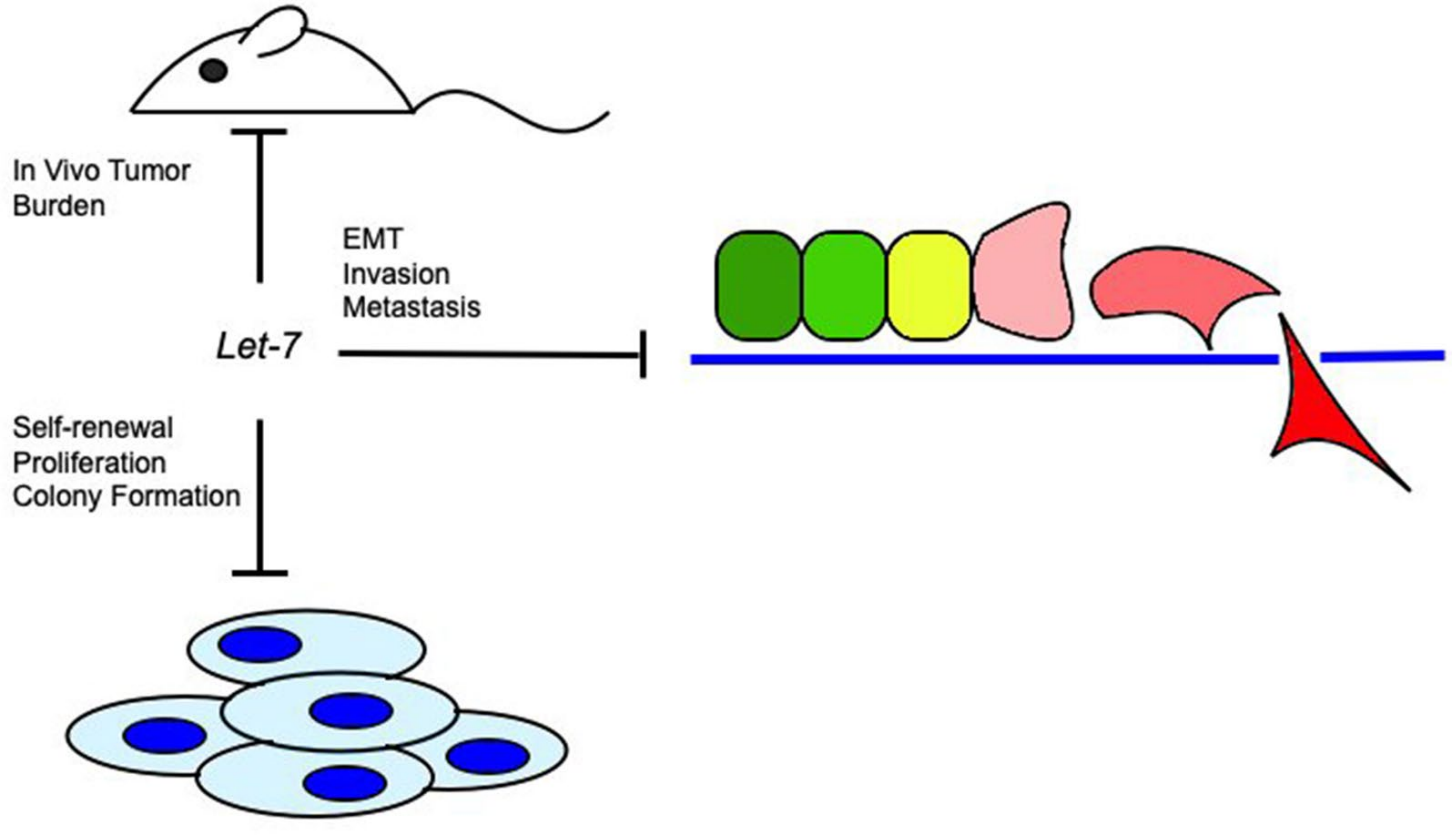
Overview of tumor-suppresive let-7 effect on cancer functional phenotype

*Let*-*7* induction of chemosensitivity seen in vitro and in vivo is due to inhibition of LIN28A/B, STAT3, E2F1, IMP1 and chemoresistance genes MDR1, ABCG2, and MMP9 [33, 37, 62, 63, 65, 89]. In EOC, *let*-*7* down-regulates BRCA1, RAD51, PARP, and IGF1, resulting in increased sensitivity to cisplatin, and longer progression free survival and overall survival [34, 59]. BRCA1, RAD51, PARP, and IGF1 proteins contribute to DNA double strand break repair, which is induced by cisplatin. Inhibiting those enzymes decreases the ability of cancer cells to survive [34, 59, 90]. Nanoparticle delivery of *let*-*7* together with paclitaxel results in an increase in sensitivity, resulting in apoptosis [59]. In blood cancers, up-regulation of the *let*-*7* family member miR-98 results in increase of BAX and p21 in acute myeloid leukemia and increased sensitivity to adriamycin [37]. Figure 2 represents overall *let*-*7* tumor suppressive function.

*Let*-*7* decreases the cellular proliferation rate due to a decreased proportion of cells in S phase of the cell cycle [56, 64, 75, 76, 91, 92]. *Let*-*7* also represses negative regulators of histone H2b monoubiquitylation (H2Bub1). H2Bub1 loss correlates with cancer progression and poor prognosis while up-regulation causes a decrease in number of breast cancer cells in S phase and cell migration [93]. Inhibition of cancer cell growth by *let*-*7* is also due to increased apoptosis via up-regulation of bak and bax and reduced bcl-xL [37, 42, 58, 60, 67]. Table 4 summarizes targets of *let*-*7* family members stating which have been reporter assay-validated.

**Table 4:** Validated and non-validated direct *Let*-*7* targets

Human *let*-*7* targets	Validated	Human *let*-*7* targets	Validated
HMGA2 [39, 57, 67, 70, 89, 142]	Yes	TARBP2 [40, 81]	Yes
HMGA1 [81]	Yes	ZC3H3 [40, 81]	Yes
LIN28A [33, 69, 115]	Yes	Etv2 [143]	Yes
c-MYC [129, 144]	Yes	Acvr1b [145]	Yes
LIN28B [33, 81, 89]	Yes	Zbtb16 (PLZF) [146]	Yes
STAT3 [63, 85]	Yes	Cyclin D1 [64, 75]	Yes
N-RAS [72, 81, 147]	Yes	Cyclin A [75]	Yes
K-RAS [147]	Yes	IMP1 [65, 89]	Yes
H-RAS [57, 147]	Yes	MAP4K3 [44]	Yes
Dicer1 [100]	Yes	ITGB3 [44]	Yes
IL-6 [119]	Yes	HIF-1A [148]	Yes
Cyclin D [35, 91]	Yes	IGF2BP1 [144, 149]	Yes
IGF1 [150]	Yes	IGF2BP2 [151]	Yes
ARID3A [97]	Yes	RSU1P2 [128]	Yes
ARID3B [80]	Yes	NEDD9 [106]	Yes
TCF-4^a^ [60]	Yes	DOCK3 [106]	Yes
MMP1 [152]	Yes	NGF [153]	Yes
NTN1 [154]	Yes	GHR [155]	Yes
INSR [115]	Yes	Twist [62]	No
IGF1R [115, 150]	Yes	Snai1 [62]	No
IRS2 [115]	Yes	Vimentin [62]	No
Pik3ip1 [115]	Yes	N-Cadherin [62]	No
AKT2 [115]	Yes	IMP2 [89]	No
TSC1 [115]	Yes	ATXN7L3 [93]	No
RICTOR [115]	Yes	USP44 [93]	No
LOX1 [156]	Yes	USP42 [93]	No
PBX3 [71]	Yes	BCL11A [157]	No
ERα [40]	Yes	TGF-βR1 [158]	No
EZH2 [35, 139]	Yes	TGF-βR3 [158]	No
E2F2 [40, 73, 81]	Yes	SMAD2 [158]	No
E2F5 [81]	Yes	FIGN [89]	No
CPSF1 [40, 81]	Yes	CDC34 [89]	No
DDX18 [40, 81]	Yes	NME6 [89]	No
EiF4A1 [40, 81]	Yes	MED6 [89]	No
EiF2C2^b^ [40, 81]	Yes	COL4A5 [89]	No
LSM6 [40, 81]	Yes	NAP1L1 [89]	No
PABPC4 [40, 81]	Yes	PIGA [89]	No
RBM38 [40, 81]	Yes	SLC25A24 [89]	No
PLAGL2 [159]	Yes	E2F1 [37]	No
AURKB [160]	Yes	E2F1 [37]	No
PLAGL2 [159]	Yes		

^a^*Let*-*7* inhibits at the promoter region

^b^*Let*-*7* increases expression

## *Let*-*7* as oncogene

Unexpectedly, *let*-*7* can also have detrimental effects. Even though *let*-*7* has been demonstrated to have tumor suppressive effects in various cancer types, emerging data suggest that, counterintuitively, in some cases *let*-*7* may act as an oncogene. Several groups have demonstrated that the *let*-*7a3* locus is highly methylated in normal tissues, but hypomethylated in lung and ovarian tumors, with *higher* expression of mature *let*-*7a* in cancers [94, 95]. Over expression of *let*-*7a3* in lung cancer cells results in increased aggressiveness of cells, assessed via anchorage independent assay and increase in gene expression associated with cell proliferation, as well as down-regulation of genes associated with adhesion, relevant to tumor progression and metastasis [95]. Higher *let*-*7a3*, *let*-*7b*, and *let*-*7c* levels in ovarian and hepatic cancers are correlated with poor prognosis and decreased overall survival [43, 94, 96]. Ma et al. demonstrated that *let*-*7e* is increased in and positively affects migration and invasion of esophageal squamous cell carcinoma cells, possibly via targeting ARID3a [97]. Since ARID3a negatively correlates with pluripotency, decreasing it could contribute to stemness [98]. *Let*-*7f* and *let*-*7e* have been shown to be upregulated in tongue squamous carcinoma, and *let*-*7c*, *let*-*7d*, and *let*-*7f* are upregulated in aggressive relative to non-aggressive tumors [99]. Mir-98 has been shown to increase chemoresistance via indirect repression of mir-152 by targeting Dicer1. Mir-152 controls RAD51 expression, contributing to the poor prognosis of EOC patients with increased levels of mir-98 [100]. In certain in vitro conditions such as starvation, *let*-*7* paradoxically induces expression of HMGA2 [101]. All of these indicate the complexity of the relationship between *let*-*7* and cancer cell aggressiveness, and illustrate the fact that the actions of any miRNA are context dependent. The set of genes expressed in a particular cell determines the available *let*-*7* targets. Thus, it is important that *let*-*7* overexpression treatment strategies be tailored towards individualized clinical scenarios based on specific miRNA expression profiles, as opposed to overarching treatment schemas spanning across multiple malignancy types.

Tumor microenvironment and stroma are also important to consider when developing new therapies. Baer et al. demonstrated that increased *let*-*7* expression in tumor associated macrophages (TAMs) results in conversion into the M2 phenotype. While tumor infiltration by TAMs with M1 phenotype have pro-inflammatory activity and better prognosis, the M2 phenotype is associated with increased angiogenesis and increased tumor burden [102]. *Let*-*7* delivery as a therapeutic regimen therefore has to be specific to cancer cells due to its oncogenic functions in tumor immune cells. Even though a few studies demonstrated *let*-*7* as having oncogenic functions and correlating with poor prognosis, the vast majority of evidence suggests otherwise. Therefore, *let*-*7* remains a potential therapeutic target.

## *Let*-*7* regulation

### Transcriptional regulation

*Let*-*7* promoters are activated by the stem cell renewal and pluripotency factor OCT-4, and are repressed by the proto-oncogene MYC, some mutant forms of p53, and in cases of cellular stress (e.g. radiation), by wild type p53 [81, 103–105]. *Let*-*7* repression by wild type p53 during stress is important when considering choice of therapy. p53 is activated by radiation, and in turn, p53 represses *let*-*7* transcription. Thus, radiotherapy could induce acquired radio-resistance stemming from the of loss of *let*-*7*. Lung tumors in which *let*-*7* levels are low correlate with low proliferation levels prior to radiotherapy. These tumors tend to exhibit accelerated reproliferation posttreatment. Thus, tumor *let*-*7* levels in lung cancer patients may inform the clinician whether radiotherapy would be counterproductive in some cases. Because p53 is involved in many cellular processes and acts differently upon different stimuli, more research is needed to study this phenomenon. The epithelial-mesenchymal transition (EMT) factor Twist also represses the *let*-*7* promoter in cooperation with BMI1 [106].

### Epigenetic regulation

Abnormal *let*-*7* expression is also due to epigenetic mechanisms. *Let*-*7* is repressed by a single nucleotide polymorphism (SNP) in the *let*-*7i* promoter region, correlating with increased susceptibility to cervical squamous cell carcinoma [107]. *Let*-*7* repression is also achieved by inhibiting *let*-*7e* promoter demethylation by JARID1B in urothelial cancer, promoter methylation by COX2/PGE2 signaling, and histone modifications of *miR*-125b in breast cancer [91, 108, 109]. *MiR*-125b and *let*-*7a2* share the same promoter, suggesting that *let*-*7a2* is repressed by this mechanism as well.

### Post-transcriptional regulation

RNA binding proteins LIN28A and LIN28B represent a major post-transcriptional *let*-*7* regulation pathway. LIN28 blocks *let*-*7* maturation with high specificity at pre- and pri- stages [110]. The cold shock domain (CSD) of Lin28 interacts with the pre-E loop, and the CCHCx2 domain with the GGAG motif at the 3′ end of *let*-*7*, inhibiting *let*-*7* processing [111]. *Let*-*7* monouridylation by terminal uridyltransferases TUT4(7) stabilizes *let*-*7* precursors for further processing, and LIN28 binding results in polyuridylation, which is a signal for degradation [112]. Figure 1 illustrates simplified *let*-*7* binding by LIN28 and TUT4(7). LIN28B represses *let*-*7* less effectively than LIN28A due to its nuclear localization, where terminal uridyltransferase, a mediator of *let*-*7* repression, is not present [113]. LIN28A is present at high levels during early embryonic development, is progressively lost as cells differentiate, and is absent in somatic cells. It aberrantly increases in cancer, repressing *let*-*7*. Elevation of LIN28 has been attributed to loss of transcriptional regulation [78].

Although these two factors, LIN28 and *let*-*7*, appear mutually exclusive, there is evidence that they can coexist. Both mature *let*-*7* and LIN28 are present in ESCs, fine-tuning each other [114]. As *let*-*7* and LIN28 co-exist in ESCs, they also coexist in normal fully differentiated cells, the balance of which is important for proper control and function, as illustrated by glucose metabolism: repression of LIN28 and *let*-*7* upregulation results in insulin resistance and impaired glucose metabolism in vivo [115]. It is also important to note that LIN28 function is not exclusively controlled by *let*-*7*. Balzer et al. demonstrated *let*-*7* independent LIN28 function during neurogliogenesis [116]. LIN28 plays an important role during terminal differentiation of mouse skeletal muscle and is detected in mouse muscle tissues, demonstrating co-expression with *let*-*7* [104, 117].

*Let*-*7* overexpression also illustrates the precise balance necessary to maintain homeostasis. While loss of *let*-*7* leads to oncogenesis, aberrantly high expression of *let*-*7* also leads to toxicity indicating that homeostasis requires a precise level of expression. Wu et al. demonstrated that *let*-*7* overexpression by 20-fold resulted in liver damage and dysfunction [66]. Based on this observation and co-expression of *let*-*7* with LIN28 in ESCs, LIN28 is considered an important regulator of *let*-*7* even in somatic cells. Furthermore, changes in LIN28 levels may alter normal cellular processes via *let*-*7* repression or up-regulation. Parisi et al. demonstrated *let*-*7* independent LIN28 increase upon exit from pluripotency [118].

Cellular signaling, including NFkB, STAT3, and MAPK-Erk pathways are also involved in *let*-*7* regulation. While MAPK-Erk signaling positively regulates *let*-*7* by inhibition of LIN28, NFkB and STAT3 cause both LIN28A up-regulation and *let*-*7* repression [85, 119, 120]. Tsanov et al. demonstrated LIN28 stabilization via phosphorylation by MAPK-Erk, which had no effect on *let*-*7* levels, in contrast to results obtained by Liu et al. showing MAPK-Erk-mediated *let*-*7* activation. The discrepancy obtained by the two groups is possibly due to differences in biology of the cell types used and experimental procedures. Liu et al. and Tsanov et al. used mouse and human embryonal carcinoma cells respectively [120, 121]; a species difference could explain the conflicting findings. While Liu et al. used a knockin LIN28 mutant to demonstrate the effect of phosphorylation, Tsanov et al. used overexpression of the mutant.

In normal cells, wild type p53 helps maintain *let*-*7* levels by disrupting the inhibitory effect of LIN28 and facilitating loading of mature *let*-*7* onto Ago2. Mutation and loss of p53 in cancer are associated with *let*-*7* repression [122, 123]. ADAR1 (adenosine deaminase acting on RNA), an RNA-binding protein, negatively regulates *let*-*7* biogenesis by altering *let*-*7* secondary structure at DROSHA and Dicer cleavage sites. ADAR1 expression is positively regulated by JAK2 signaling, and is overexpressed in CML and presumably in other cancers where JAK2 signaling is increased [124]. *Let*-*7* is also inhibited post-transcriptionally by (DCAMKL-1) in colorectal cancer [125].

Aside from repression at the level of transcription and post-transcriptional processing, other RNAs can also inhibit *let*-*7*. MiR-107 forms complexes with *let*-*7* and increases its degradation [126]. Long non-coding (lnc) RNA H19, *linc*-*ROR*, *CCR*492, and lnc *RSU1P2* inhibit *let*-*7* function by acting as sponges. MiR-107 is over-expressed in some breast cancers, *linc*-*ROR* in pancreatic ductal adenocarcinoma, and lnc *RSU1P2* in cervical cancer, where they contribute to cancer progression and poor prognosis [49, 127–129]. In glioblastoma, insulin-like growth factor 2 binding protein 2 (IMP2) blocks *let*-*7* function by binding to miRNA recognition elements of *let*-*7* targets. There is a lack of negative correlation between *let*-*7* and its targets in spheroids due to the protective effect of IMP2. In cancers expressing IMP2, its repression may be necessary together with *let*-*7* up-regulation to obtain the desired tumor suppressive effect [130]. Table 5 lists factors that regulate *let*-*7* at transcriptional, post-transcriptional, and functional levels.

**Table 5:** *Let*-*7* regulators on transcriptional, post-transcriptional, and functional levels

*Let*-*7* regulation			
Inhibitor	Family member	Context	Mechanism
JARID1B [91]	*let*-*7e*	Breast cancer	
p53 mutant [81]	*let*-*7i*	Lung cancer	
DCMAKL-1 [125]	*let*-*7a*	Colorectal cancer	
MYC [105]	*let*-*7a*-*1*, *f*-*1*, *d*	Hepatocellular carcinoma	
OCT-1 [156]	*let*-*7g*	Aorta smooth muscle cells	
COX2* [161]	*let*-*7b*	Urothelial cancer	
TWIST [106]	*let*-*7i*	Head and neck cancer	
BMI1 [106, 162]	*let*-*7i*	Head and neck cancer	
KDM2B [163]	*let*-*7b*	Embryonic fibroblasts	Promoter methylation
LIN28 [110, 113, 133]	*let*-*7a1*, *a2*, *b*, *c*, *d*, *e*, *g*, *f1*, *f2*, *I*, *Mir*-*98*	Mouse ESCs, Hela cells	
STAT3 [85]	*let*-*7a*	Ewing sarcoma	NFkB activation
NFkB [119]	*let*-*7a*, *b*, *c*, *d*, *f*	Breast cancer	LIN28 up-regulation
*MiR*-*107* [126]	*let*-*7a*	Breast cancer	
*LncRNA H19* [49]	*let*-*7a*, *b*	Breast cancer	
*Link*-*ROR* [127]	*let*-*7i*, *b*, *e*, *c*	Pancreatic cancer	
IMP2 [130]	*Seed*	Glioblastoma stem cells	Target stabilization
LncRNA *RSU1P2* [128]	*let*-*7a*	Cervical caner	
ADAR1 [124]	*let*-*7d*	Leukemia stem cells	
LncRNA *CCR492* [129]	*Seed*	Mouse embryonic fibroblast	
eEBPa [164]	*let*-*7a2*	Lung cancer	
SNP rs10877887 [107]	*let*-*7i*	Cervical cancer	
P53 [104]	*let*-*7a*, *b*	Colon cancer	Cellular stress

Activator	Family member	Context	
ZEB1 [97]	*let*-*7e*	Esophageal cancer	
OCT4 [103]	*let*-*7a*-*2*	Cervical cancer	
NF-kB [165]	*let*-*7a*-*3/b*	HeLa, 293T	
ESE3/EHF [78]	*let*-*7b*	Prostate cancer	
P53 [122]	*let*-*7 a*, *b*, *c*, *e*, *f*, *g*, *i*, *Mir*-*98*	Colon cancer	
Tritetraspolin [123]	*let*-*7b*, *f*	Ovarian cancer	
MAPK-Erk [120]	*let*-*7a*, *g*	Mouse embryonic carcinoma	

*Indirect by inducing promoter methylation

## Differences between individual family members

### Functional differences

Since mature miRNA *let*-*7* family members have nearly identical sequences, in general, it is assumed that they function similarly and have common targets, due to off target binding for which miRNAs are notorious. However, there is some evidence that different members of the *let*-*7* family do have different functions, most likely due to unique target preferences, and therefore cannot be considered as one. In hepatocellular carcinoma, it has been demonstrated that overexpression of different *let*-*7* family members affects cell viability to different extents: *let*-*7a* has the greatest effect [60]. It has been demonstrated that when over-expressed together, *let*-*7i* and *let*-*7g* had a greater effect on hepatoma cell division and apoptosis than overexpression of individual miRNAs, suggesting that members of this family may act in synergy to deliver tumor suppressive actions and other physiological functions [131]. Takamizawa et al. demonstrated that while both *let*-*7a* and f reduce the ability of lung cancer to form colonies, *let*-*7f* is able to do so to a greater extent [47].

### Regulatory differences

Since *let*-*7* family members are located in different clusters, transcriptional regulation is different in each case. During neural differentiation, *let*-*7a1*, *a2*, *d*, *f2*, and *i* are active in several cell types and constitutively transcribed, while *let*-*7a3*, *b*, *c*, *e*, and *g* show dynamic transcription. This difference may be due to the number of transcription start sites (TSS) present in their promoter regions. Multiple TSS produces dynamic expression because more transcription factors are involved in regulation [132]. Another way *let*-*7* family members differ from each other is via post-transcriptional regulation. One study has demonstrated that miR-107, which contributes to metastasis of breast cancer by inhibiting *let*-*7*, binds to different *let*-*7* members with different efficiency [126]. Different *let*-*7* family members are repressed by LIN28 to different degrees, and in fact *let*-*7a3* bypasses repression by LIN28 altogether due to a different sequence in the preE region of the bulge [133].

*Let*-*7* over-expression has been widely investigated as a therapeutic agent to inhibit progression of many cancers in vitro and in animal models. It is important to consider which mature *let*-*7* family member would be the most beneficial to patient survival before developing it into therapy.

## Conclusion and future direction

In this review, we emphasize the importance of miRNA *let*-*7* in cancer. We focus on the potential to use *let*-*7* in precision medicine for screening and diagnosis of cancer, for its prognostic value, and as a therapeutic agent. We review the complex regulation and function of the *let*-*7* family members, and focus on their abnormal regulation in cancer, which leads to abnormal and/or loss of function. *let*-*7* miRNAs have been referred to as tumor suppressors, but it is important to consider that there is evidence to support their oncogenic functions in vitro and in clinical subjects. Our goal is to demonstrate the importance of *let*-*7* during treatment decisions for chemo- and radiotherapy, to enable its use as precision medicine, and to deliver optimal results for patients.

*Let*-*7* remains a promising cancer therapy and warrants more research; but even before all details of its therapeutic use are worked out, tumor *let*-*7* levels can be used to choose the best therapy options for each individual. Low or high tumor *let*-*7* levels can point to the most effective therapy regimens, and its levels in bodily fluids show potential for use as an aid to diagnosis, therapy monitoring, and prognosis.

Many questions remain unanswered. Knowledge of levels of all *let*-*7* family members in each type of cancer can provide a more precise overview of its regulation, and provide more specific diagnostic/prognostic tools. Functional studies may reveal that upregulation of a specific *let*-*7* member offers the most beneficial effect as a therapeutic regimen. Combination of standard therapy with *let*-*7* over-expression has to be well studied in order to avoid toxicity and unwanted interactions. More in vivo models are needed to develop *let*-*7* into a safe and effective therapy regimen that will provide the rationale for clinical trials.

## Data Availability

Data sharing is not applicable to this article as no datasets were generated or analyzed during the current study.

## Abbreviations

ABCG2: ATP Binding Cassette Subfamily G Member 2
ADAR1: adenosine deaminase 1
AGO2: argonaute 2
Akt: protein kinase B
ARID3A: AT-rich interaction domain 3A
BAX: BCL2 associated X protein, apoptosis regulator
BCL-xL: apoptosis inhibiting protein
BRCA1/2: DNA repair associated protein 1/2
CCR492: cell-cycle regulated long non-coding RNA
CDK4: cyclin dependent-kinase 4
COX2: stem cell factor
CSC: cancer stem cell
CSD: cold shock domain
DCAMKL-1: doublecortin and CaM kinase-like 1
DICER: ribonuclease
EMT: epithelial-to-mesenchymal transition
EOC: epithelial ovarian cancer
ESC: embryonic stem cell
GHR: growth hormone receptor
H2Bub1: histone H2B monoubiquitylation
HMGA2: high mobility group AT-Hook 2
IGF1: insulin like growth factor 1
IGF1R: insulin like growth factor 1 receptor
IGF2BP1: insulin like growth factor 2 binding protein 1
IGF2BP2: insulin like growth factor 2 binding protein 2
IMP2: insulin-like growth factor 2 binding protein 2
JAK2: janus kinase 2
JARID1B: jumonji AT-rich interactive domain 1B
LIN28A/B: LIN28 homolog A/B
Lnc H19: long non-coding RNA H19
Lnc ROR: long non-coding RNA regulator of reprogramming
Lnc RSU1P2: long non-coding RNA Ras suppressor protein 1 pseudogene 2
MDR1: multi-drug resistance-1
MIRLET7: miRNA *lethal*-*7*
miRNA: microRNAs
MMP1: matrix metallopeptidase 1
MMP9: matrix metallopeptidase 9
MYC: proto-oncogene protein
N-Cadherin: neuronal cadherin
NGF: nerve growth factor
NTN1: netrin-1
OCT-4: octamer-4 embryonic gene
P21: cell cycle regulator
PARP1/2: poly (ADP-ribose) polymerase 1/2
PEG: polyethyleneglycol
PGE2: prostaglandin E2
PLAGL2: pleiomorphic adenoma gene-like 2
RAD51: DNA repair protein
SNP: single nucleotide polymorphism
STAT3: signal transducer and activator of transcription-3
TAM: tumor associated macrophage
TCF-4: transcription factor 4
TSS: transcription start site
TUT4(7): terminal uridylyl transferase 4(7)
WNT: signaling pathway
